# A workshop report on promoting HIV/AIDS understanding through a capacity building train-the-trainer educational intervention

**DOI:** 10.4314/pamj.v10i0.72226

**Published:** 2011-10-10

**Authors:** Holly J Diesel, Dickson S Nsagha, Clement M Sab, Donna Taliaferro, Neal S Rosenburg

**Affiliations:** 1 Goldfarb School of Nursing at Barnes Jewish College, 4483 Duncan, St. Louis, Missouri, USA; 2 Department of Public Health and Hygiene, Faculty of Health Sciences, University of Buea, PO Box 63, Buea, Cameroon; 3 Goldfarb School of Nursing at Barnes Jewish College, 4483 Duncan, St. Louis, Missouri, USA; 4 Linfield College, 900 Baker Street, McMinnville, Oregon, 97128, USA

**Keywords:** Workshop, HIV, AIDS, capacity building, education, intervention, Cameroon

## Abstract

Nursing educators are frequently confronted with challenges that bring about innovation and transition to new ways of transferring knowledge in their home environments. These challenges are magnified when approached from an international perspective. Optimal implementation of knowledge transfer incorporates choosing models that promote local initiatives in line with increasingly decentralized educational structures. These decentralized models are a means to foster ongoing participation for both educators and students in their own professional development. Innovative education stems from creativity in approaching the need with formats and activities to meet a specific challenge. This experimental study builds upon previous study by the authors which was conducted in March, 2009, based upon the qualitative open focus forum at each of the five nursing programs. Overwhelmingly, the Cameroonian nursing students expressed a keen desire to study the HIV infected pregnant woman and the feeding options of the newborn. The study team developed the train-the-trainer program which was delivered at the University of Buea in the Southwest region of Cameroon in March, 2011. TTT is particularly effective for reaching large audiences and also permits a degree of sustainability such that the Cameroonian students will be trainers for subsequent cohorts of their peers. This study continues to strengthen the collaborative endeavors between the two nursing schools; the University of Buea (UB) and Goldfarb School of Nursing (GSON) at Barnes Jewish College in Saint Louis, Missouri, USA. The final aim of the intervention was the initiation of collaborative relationships between the faculty members of the two educational organizations.

## Introduction

Implementing transformative and sustainable change in developing nations requires long-term commitment to the reform of systems and the development of resources. Increasing attention has been given to gender issues, in particular to women's sexual and reproductive health. Despite this attention, the health problems of women in developing countries remain a low priority [1]. Recent research has shown the importance of addressing attitudinal change in the practices of nurses caring for people living with HIV/AIDS (PLWHA) [2,3]. Nursing partnerships established to build capacity can be an important resource, especially when considering nurses’ pivotal role in generating and transferring knowledge to students, who will eventually address complex changes in health care. Notably, the workshop planned in Cameroon was founded upon the evidence that African women's health care needs have been largely ignored [4]. One issue of particular importance in Africa specifically in Cameroon is that of HIV/AIDS in obstetrical and newborn populations. HIV infection is a condition that carries a high level of stigmatization [5–7]. In order to attain effective care giving of the mother/infant dyad, properly educating women is an essential component [8]. However, that assumes that there are individuals equipped with the correct information to teach the women. In a climate where people living with HIV/AIDS (PLWHA) are often blamed for their condition and transmission of HIV is poorly understood by many people in the general population, knowledge transfer is a critical aspect to break the cycle of infection. In 2009, two faculty members from the Goldfarb School of Nursing at Barnes Jewish College (GSON) traveled to Cameroon to assess knowledge, attitudes and beliefs regarding HIV-related stigma of nursing students at five colleges of nursing in Buea [2]. Following the surveys, open forums were held where students voiced their desire for more information specific to the care of obstetrical and newborn populations with HIV.

Peer-centered and train-the-trainer educational models suggest positive and lasting educational outcomes in the nursing environment. The peer-leader model provides immediate problem-solving assistance to potential clinical inquiries. Additionally, the U.S. peer-leaders developed extended mentoring relationships that nurtured the Cameroonian peer-leaders via e-mail communication post educational workshop [9]. This project is based on the Centering Model which included ten 90-minute, small group, peer-lead, educational sessions that offer a non-rigid, flexible discussion format for learning [10]. There are essential elements of the Centering Model; one such component requires that the peer-leader assume a facilitative role rather than traditional lecture style [11]. The principal activities to meet the program outcomes include: a) Peer-training in which U.S. nursing students collaboratively conceptualize the underpinnings and deliver strategies for obstetric nursing and HIV, b) multiple educational formats for content delivery such as cultural student immersion, c) ten sessions of 90-minute theory driven women-infant nursing care with the identification of country specific action plans (delivered in-class lectures by Rosenburg and Diesel) , d) establish intercontinental community relationships for continued educational endeavors based upon the needs expressed by the local communities.

A lack of cultural competencies exhibited by nurses clearly threatens to alienate the patients whom they intend to help. The effects of international education infer that transition and adaptation to another culture are an effective manner for students to develop a deeper understanding of self and cultural sensitivity [12]. This lack of preparation often leaves the nurse with a feeling of despair and ill-prepared when providing care to HIV-infected and affected populations within their own communities. Roland [13] suggests that undergraduate nursing programs have a responsibility to more adequately prepare nursing students to become more culturally aware in an increased effort to satisfy the demands of patients from various cultural backgrounds. Previous studies have shown that students who participated in global immersion educational programs demonstrated significantly more cognitive growth than those who did not. Additionally, nursing students overcame their ethnocentrism and gained valuable personal, interpersonal and global long-term effects [14].

## Workshop report

### Aim of the workshop

The aim of the workshop was to transfer knowledge related to the care of pregnant women and newborns infected with HIV by: 1) providing students with small pods led by a peer nursing students, 2) providing students with leadership opportunities, 3) impacting attitudes and understanding in culturally congruent setting, 4) providing opportunities for further contact between nursing students in a global setting, 5) building capacity, 6) establishing ongoing partnerships between the two campuses.

### Participants

The workshop was delivered in a train-the-trainer format. Eight senior nursing students from the United States from GSON provided the content daily over a four-day workshop was held in early March of 2010 for 52 University of Buea (UB) nursing students from levels 300 and 400. Each day lasted approximately eight hours. All students spoke and read English as a primary language.

### Facilitators

Facilitators and organizers were chosen from the two schools of nursing for their content expertise and ability to plan and coordinate the event. HJD is an associate professor of nursing at GSON with over 20 years obstetrical/newborn experience. NDS a lecturer at the Faculty of Health Sciences of the University of Buea in Cameroon who has been working on HIV/AIDS for many papers. NSR is an associate dean of nursing at Linfield College whose research focuses on HIV-related stigma. SCM is a nursing lecturer at the University of Buea and a seasoned nurse specialized in nutrition and community interventions. DT is a Nursing Professor and Associate Dean for Research and Paul J. McKee, Jr. Endowed Professor at the Goldfarb School of Nursing who has researched on HIV for over two decades. PE is an Assistant Professor and Biostatistician at GSON with five years of experience managing and analyzing data. The two primary sponsors were Professor Michael Evans, Dean of Goldfarb School of Nursing at Barnes Jewish College and Professor Vincent Titanji, Vice-Chancellor of the University of Buea.

### Pre-workshop tasks

Six months prior to the workshop, the facilitators collaborated to identify content and delivery methodology. The GSON facilitators selected the US students to travel to Cameroon from the roster of students taking an HIV elective course, and then worked with them to develop the presentation. In addition, the GSON facilitators obtained IRB approval in the USA. Five surveys were selected to measure changes in attitudes, beliefs and knowledge in a longitudinal format. These surveys were designed to measure the impact of the workshop on the UB nursing students, when delivered by their US peer nursing students. The Cameroonian facilitators identified UB students and obtained ethical clearance from the local authorities, as well as organizing local logistics.

### Program

The four-day workshop was delivered in a train-the-trainer format. This was done in small pods of 7 to 8 UB nursing students with one GSON nursing student in the role of lead trainer. Each pod met for three hours in the morning, followed by lunch and then three additional hours in the afternoon. Prior to the delivery of any content, all UB nursing students completed surveys to measure knowledge, attitudes and beliefs regarding HIV. Each student had their own set of content handouts, and each pod had one text book, The Person Living with HIV/AIDS: Nursing Perspectives (Durham & Lashley, 2010) for reference. Participation was interactive and all presentations were done in English. Participants covered the following content: history, epidemiology, trajectory of the disease, ethics of care, medical management, nursing management of symptoms, medications, nutrition, ante-partum/intrapartum/postpartum and newborn care, and post birth feeding options. At the conclusion of content, the UB students completed the surveys again, in order to measure changes in knowledge, attitudes and beliefs. The reference textbooks, as well as a variety of medical and nursing texts were donated to the UB at the end of the workshop. Table 1 depicts the sequencing of all topics covered during the course of the workshop.

**Table 1:** Content presented during four-day train the trainer workshop

**Day 1**	Orientation and Introductions Pretest survey Pod identification Tour of University of Buea

**Day 2**	Etiology, Epidemiology, Transmission of HIV HIV Screen, Testing and Counseling Disease Trajectory Ethical and Legal Dimensions of HIV Prevention of HIV
**Day 3**	Medical Management of HIV Managing Symptoms of HIV Living with HIV/AIDS Caregivers of People Living with HIV Nursing Case Management within the Global Community Conspiracy Theories of Transmission
**Day 4**	Antepartum Care of Patients with HIV Intrapartum Care of Patients with HIV Postpartum Care of Patients with HIV HIV in Newborns Feeding Options Posttest survey Posttest survey

### Evaluation

The workshop was evaluated after the closing ceremony and the distribution of student certificates of workshop completion. An open forum was held to obtain feedback from all American and Cameroonian students who participated in the workshop led by one of the facilitators. The major themes identified in the evaluation form are listed in Table 2. Changes in knowledge, attitudes and beliefs were measured using the following surveys: 1) AIDS Attitude Scale, 2) AIDS Knowledge Scale, 3) HASI-NS, HIV/AIDS Stigma Instrument-Nursing Student, 4) Willingness to Provide Care Scale, and 50 Obstetrical HIV/AIDS Knowledge Scale.

**Table 2:** Evaluation comments from student participants

**Strengths**	Open discussion facilitated sharing Personal stories demonstrated Small group size facilitated learning Americans didn't act as if they knew more, felt like equals Impressed with the mastery of the subject Zeal of the students was inspirational Liked being in an informal setting Impressed that American faculty circulated with students
**Opportunities for improvement**	Wanted the workshop to be longer than just 4 days More time for social and cultural events Schedule all lunch times together Americans wanted to know more about local HIV care strategies Cameroonian students wanted to have the opportunity to present
